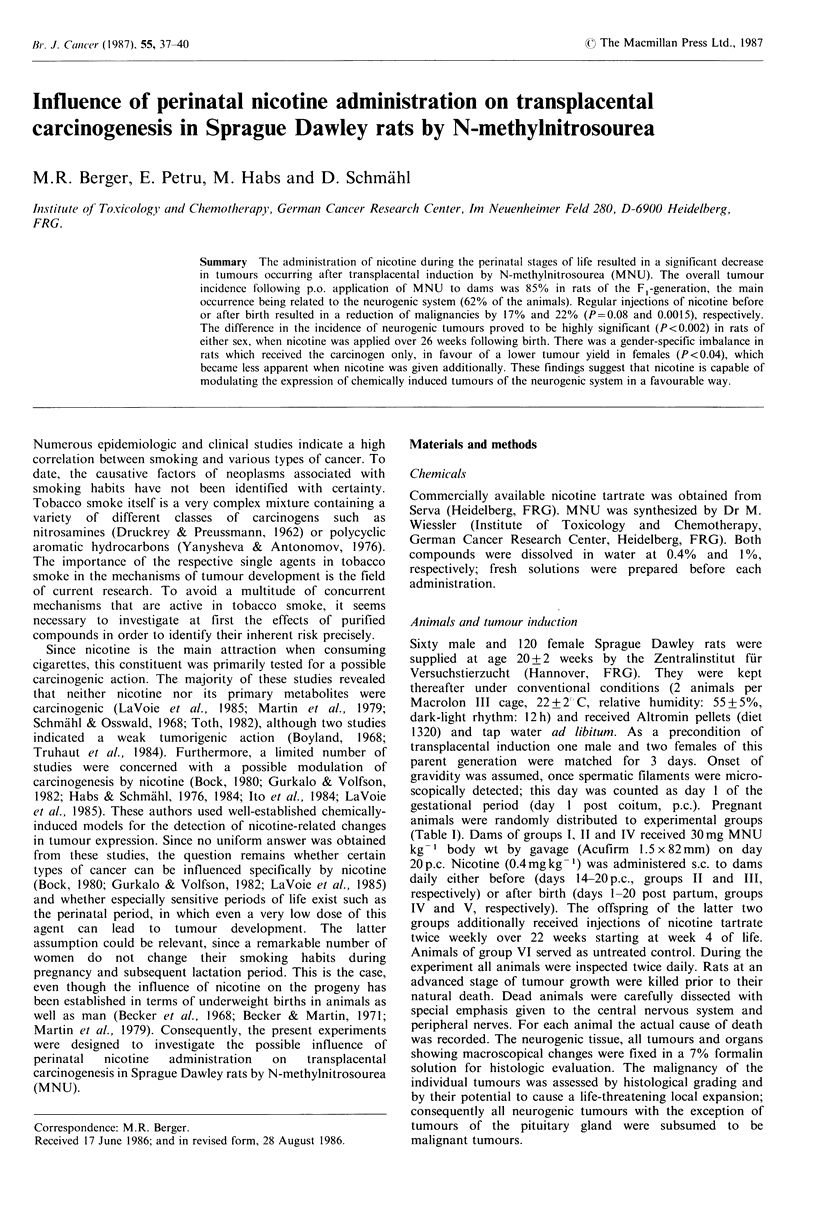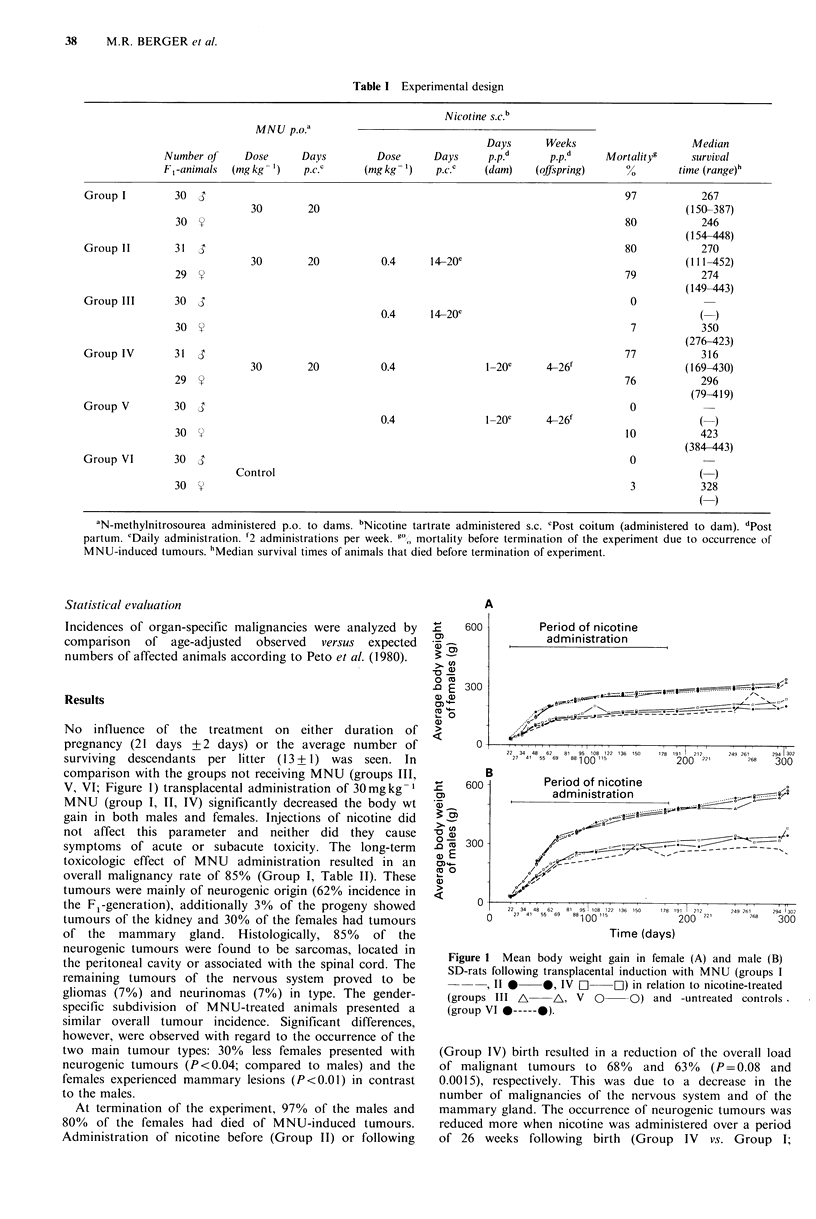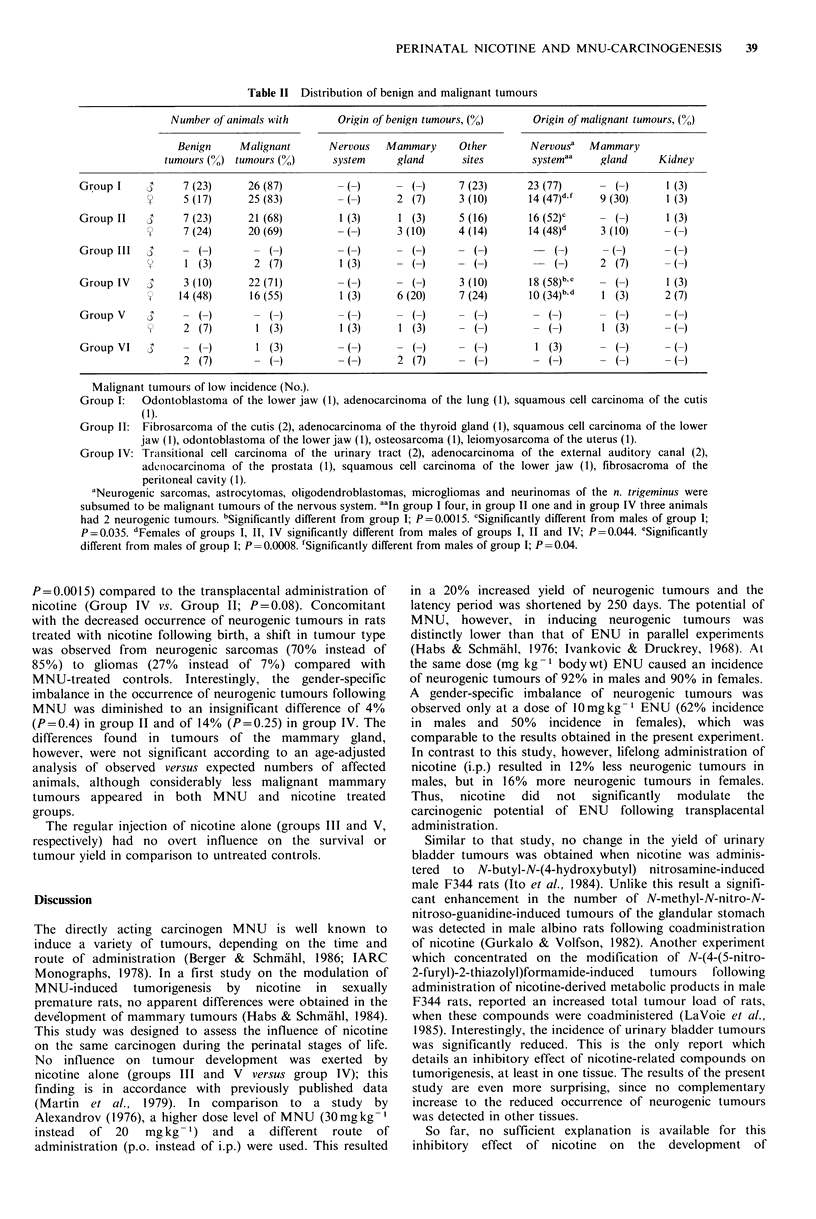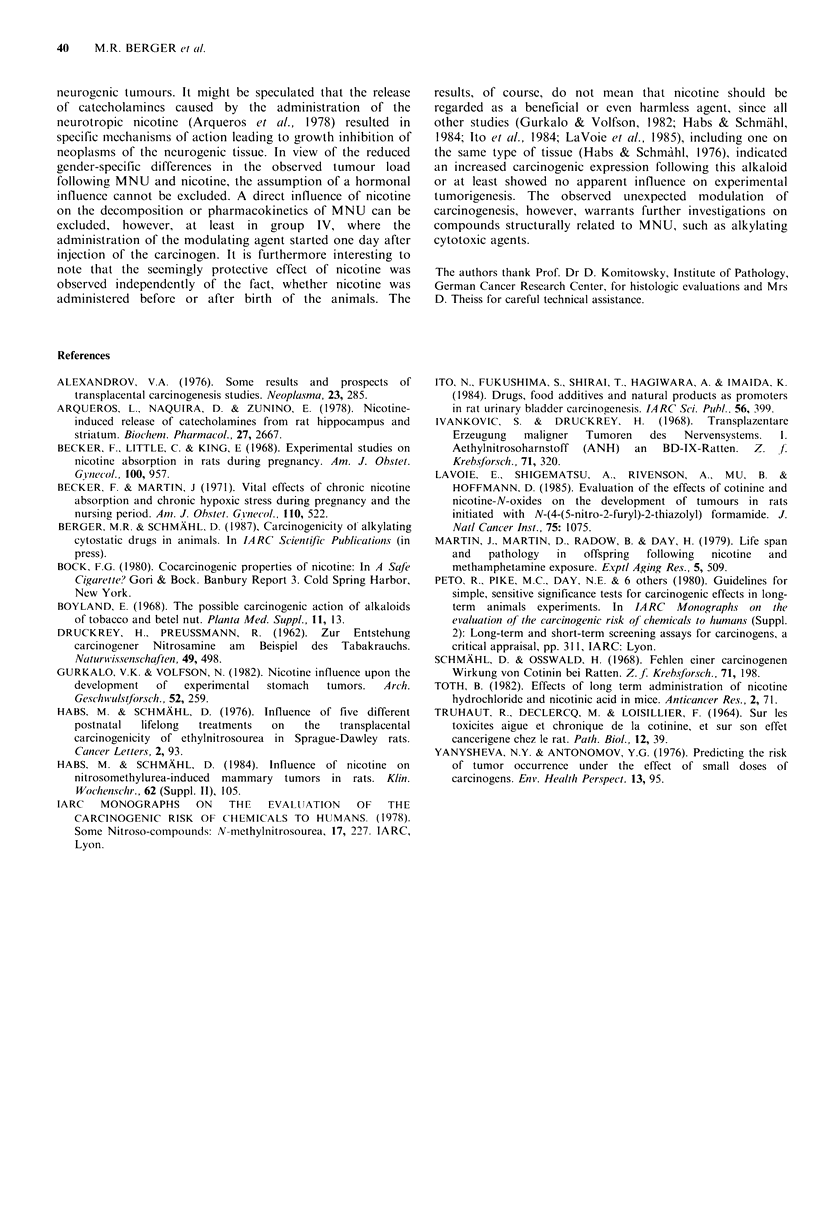# Influence of perinatal nicotine administration on transplacental carcinogenesis in Sprague Dawley rats by N-methylnitrosourea.

**DOI:** 10.1038/bjc.1987.8

**Published:** 1987-01

**Authors:** M. R. Berger, E. Petru, M. Habs, D. Schmähl

## Abstract

The administration of nicotine during the perinatal stages of life resulted in a significant decrease in tumours occurring after transplacental induction by N-methylnitrosourea (MNU). The overall tumour incidence following p.o. application of MNU to dams was 85% in rats of the F1-generation, the main occurrence being related to the neurogenic system (62% of the animals). Regular injections of nicotine before or after birth resulted in a reduction of malignancies by 17% and 22% (P = 0.08 and 0.0015), respectively. The difference in the incidence of neurogenic tumours proved to be highly significant (P less than 0.002) in rats of either sex, when nicotine was applied over 26 weeks following birth. There was a gender-specific imbalance in rats which received the carcinogen only, in favour of a lower tumour yield in females (P less than 0.04), which became less apparent when nicotine was given additionally. These findings suggest that nicotine is capable of modulating the expression of chemically induced tumours of the neurogenic system in a favourable way.


*Br. J. Cancer* (1987), **55**, 37–40 © The Macmillan Press Ltd., 1987

### Influence of perinatal nicotine administration on transplacental carcinogenesis in Sprague Dawley rats by N-methylnitrosourea

M.R. Berger, E. Petru, M. Habs and D. Schmähl

*Institute of Toxicology and Chemotherapy, German Cancer Research Center, Im Neuenheimer Feld 280, D-6900 Heidelberg, FRG.*

**Summary** The administration of nicotine during the perinatal stages of life resulted in a significant decrease in tumours occurring after transplacental induction by N-methylnitrosourea (MNU). The overall tumour incidence following p.o. application of MNU to dams was 85% in rats of the $F_1$-generation, the main occurrence being related to the neurogenic system (62% of the animals). Regular injections of nicotine before or after birth resulted in a reduction of malignancies by 17% and 22% ($P=0.08$ and 0.0015), respectively. The difference in the incidence of neurogenic tumours proved to be highly significant ($P<0.002$) in rats of either sex, when nicotine was applied over 26 weeks following birth. There was a gender-specific imbalance in rats which received the carcinogen only, in favour of a lower tumour yield in females ($P<0.04$), which became less apparent when nicotine was given additionally. These findings suggest that nicotine is capable of modulating the expression of chemically induced tumours of the neurogenic system in a favourable way.

Numerous epidemiologic and clinical studies indicate a high correlation between smoking and various types of cancer. To date, the causative factors of neoplasms associated with smoking habits have not been identified with certainty. Tobacco smoke itself is a very complex mixture containing a variety of different classes of carcinogens such as nitrosamines (Druckrey & Preussmann, 1962) or polycyclic aromatic hydrocarbons (Yanysheva & Antonomov, 1976). The importance of the respective single agents in tobacco smoke in the mechanisms of tumour development is the field of current research. To avoid a multitude of concurrent mechanisms that are active in tobacco smoke, it seems necessary to investigate at first the effects of purified compounds in order to identify their inherent risk precisely.

Since nicotine is the main attraction when consuming cigarettes, this constituent was primarily tested for a possible carcinogenic action. The majority of these studies revealed that neither nicotine nor its primary metabolites were carcinogenic (LaVoie *et al.*, 1985; Martin *et al.*, 1979; Schmähl & Osswald, 1968; Toth, 1982), although two studies indicated a weak tumorigenic action (Boyland, 1968; Truhaut *et al.*, 1984). Furthermore, a limited number of studies were concerned with a possible modulation of carcinogenesis by nicotine (Bock, 1980; Gurkalo & Volfson, 1982; Habs & Schmähl, 1976, 1984; Ito *et al.*, 1984; LaVoie *et al.*, 1985). These authors used well-established chemically-induced models for the detection of nicotine-related changes in tumour expression. Since no uniform answer was obtained from these studies, the question remains whether certain types of cancer can be influenced specifically by nicotine (Bock, 1980; Gurkalo & Volfson, 1982; LaVoie *et al.*, 1985) and whether especially sensitive periods of life exist such as the perinatal period, in which even a very low dose of this agent can lead to tumour development. The latter assumption could be relevant, since a remarkable number of women do not change their smoking habits during pregnancy and subsequent lactation period. This is the case, even though the influence of nicotine on the progeny has been established in terms of underweight births in animals as well as man (Becker *et al.*, 1968; Becker & Martin, 1971; Martin *et al.*, 1979). Consequently, the present experiments were designed to investigate the possible influence of perinatal nicotine administration on transplacental carcinogenesis in Sprague Dawley rats by N-methylnitrosourea (MNU).

Correspondence: M.R. Berger.
Received 17 June 1986; and in revised form, 28 August 1986.

### Materials and methods

### *Chemicals*

Commercially available nicotine tartrate was obtained from Serva (Heidelberg, FRG). MNU was synthesized by Dr M. Wiessler (Institute of Toxicology and Chemotherapy, German Cancer Research Center, Heidelberg, FRG). Both compounds were dissolved in water at 0.4% and 1%, respectively; fresh solutions were prepared before each administration.

### *Animals and tumour induction*

Sixty male and 120 female Sprague Dawley rats were supplied at age 20±2 weeks by the Zentralinstitut für Versuchstierzucht (Hannover, FRG). They were kept thereafter under conventional conditions (2 animals per Macrolon III cage, 22±2°C, relative humidity: 55±5%, dark-light rhythm: 12h) and received Altromin pellets (diet 1320) and tap water *ad libitum*. As a precondition of transplacental induction one male and two females of this parent generation were matched for 3 days. Onset of gravidity was assumed, once spermatic filaments were microscopically detected; this day was counted as day 1 of the gestational period (day 1 post coitum, p.c.). Pregnant animals were randomly distributed to experimental groups (Table I). Dams of groups I, II and IV received 30 mg MNU $kg^{-1}$ body wt by gavage (Acufirm 1.5×82 mm) on day 20 p.c. Nicotine (0.4 mg $kg^{-1}$) was administered s.c. to dams daily either before (days 14–20 p.c., groups II and III, respectively) or after birth (days 1–20 post partum, groups IV and V, respectively). The offspring of the latter two groups additionally received injections of nicotine tartrate twice weekly over 22 weeks starting at week 4 of life. Animals of group VI served as untreated control. During the experiment all animals were inspected twice daily. Rats at an advanced stage of tumour growth were killed prior to their natural death. Dead animals were carefully dissected with special emphasis given to the central nervous system and peripheral nerves. For each animal the actual cause of death was recorded. The neurogenic tissue, all tumours and organs showing macroscopical changes were fixed in a 7% formalin solution for histologic evaluation. The malignancy of the individual tumours was assessed by histological grading and by their potential to cause a life-threatening local expansion; consequently all neurogenic tumours with the exception of tumours of the pituitary gland were subsumed to be malignant tumours.

**Table I** Experimental design

| | Number of $F_1$-animals | MNU p.o.[a] Dose ($mg\,kg^{-1}$) | MNU p.o.[a] Days p.c.[c] | Nicotine s.c.[b] Dose ($mg\,kg^{-1}$) | Nicotine s.c.[b] Days p.c.[c] | Nicotine s.c.[b] Days p.p.[d] (dam) | Nicotine s.c.[b] Weeks p.p.[d] (offspring) | Mortality[g] % | Median survival time (range)[h] |
|---|---|---|---|---|---|---|---|---|---|
| Group I | 30 ♂ | 30 | 20 | | | | | 97 | 267 (150–387) |
| | 30 ♀ | | | | | | | 80 | 246 (154–448) |
| Group II | 31 ♂ | 30 | 20 | 0.4 | 14–20[e] | | | 80 | 270 (111–452) |
| | 29 ♀ | | | | | | | 79 | 274 (149–443) |
| Group III | 30 ♂ | | | 0.4 | 14–20[e] | | | 0 | — (—) |
| | 30 ♀ | | | | | | | 7 | 350 (276–423) |
| Group IV | 31 ♂ | 30 | 20 | 0.4 | | 1–20[e] | 4–26[f] | 77 | 316 (169–430) |
| | 29 ♀ | | | | | | | 76 | 296 (79–419) |
| Group V | 30 ♂ | | | 0.4 | | 1–20[e] | 4–26[f] | 0 | — (—) |
| | 30 ♀ | | | | | | | 10 | 423 (384–443) |
| Group VI | 30 ♂ | Control | | | | | | 0 | — (—) |
| | 30 ♀ | | | | | | | 3 | 328 (—) |

[a]N-methylnitrosourea administered p.o. to dams. [b]Nicotine tartrate administered s.c. [c]Post coitum (administered to dam). [d]Post partum. [e]Daily administration. [f]2 administrations per week. [g]% mortality before termination of the experiment due to occurrence of MNU-induced tumours. [h]Median survival times of animals that died before termination of experiment.

*Statistical evaluation*

Incidences of organ-specific malignancies were analyzed by comparison of age-adjusted observed *versus* expected numbers of affected animals according to Peto *et al.* (1980).

### Results

No influence of the treatment on either duration of pregnancy (21 days $\pm 2$ days) or the average number of surviving descendants per litter ($13 \pm 1$) was seen. In comparison with the groups not receiving MNU (groups III, V, VI; Figure 1) transplacental administration of $30\,mg\,kg^{-1}$ MNU (group I, II, IV) significantly decreased the body wt gain in both males and females. Injections of nicotine did not affect this parameter and neither did they cause symptoms of acute or subacute toxicity. The long-term toxicologic effect of MNU administration resulted in an overall malignancy rate of 85% (Group I, Table II). These tumours were mainly of neurogenic origin (62% incidence in the $F_1$-generation), additionally 3% of the progeny showed tumours of the kidney and 30% of the females had tumours of the mammary gland. Histologically, 85% of the neurogenic tumours were found to be sarcomas, located in the peritoneal cavity or associated with the spinal cord. The remaining tumours of the nervous system proved to be gliomas (7%) and neurinomas (7%) in type. The gender-specific subdivision of MNU-treated animals presented a similar overall tumour incidence. Significant differences, however, were observed with regard to the occurrence of the two main tumour types: 30% less females presented with neurogenic tumours ($P<0.04$; compared to males) and the females experienced mammary lesions ($P<0.01$) in contrast to the males.

At termination of the experiment, 97% of the males and 80% of the females had died of MNU-induced tumours. Administration of nicotine before (Group II) or following

**Figure 1** Mean body weight gain in female (A) and male (B) SD-rats following transplacental induction with MNU (groups I ———, II ●——●, IV □——□) in relation to nicotine-treated (groups III △——△, V ○——○) and -untreated controls (group VI ●-----●).

(Group IV) birth resulted in a reduction of the overall load of malignant tumours to 68% and 63% ($P=0.08$ and 0.0015), respectively. This was due to a decrease in the number of malignancies of the nervous system and of the mammary gland. The occurrence of neurogenic tumours was reduced more when nicotine was administered over a period of 26 weeks following birth (Group IV *vs.* Group I;

**Table II** Distribution of benign and malignant tumours

| | | *Number of animals with* | | *Origin of benign tumours, (%)* | | | *Origin of malignant tumours, (%)* | | |
|---|---|---|---|---|---|---|---|---|---|
| | | *Benign tumours (%)* | *Malignant tumours (%)* | *Nervous system* | *Mammary gland* | *Other sites* | *Nervous[a] system[aa]* | *Mammary gland* | *Kidney* |
| Group I | ♂ | 7 (23) | 26 (87) | - (-) | - (-) | 7 (23) | 23 (77) | - (-) | 1 (3) |
| | ♀ | 5 (17) | 25 (83) | - (-) | 2 (7) | 3 (10) | 14 (47)[d,f] | 9 (30) | 1 (3) |
| Group II | ♂ | 7 (23) | 21 (68) | 1 (3) | 1 (3) | 5 (16) | 16 (52)[c] | - (-) | 1 (3) |
| | ♀ | 7 (24) | 20 (69) | - (-) | 3 (10) | 4 (14) | 14 (48)[d] | 3 (10) | - (-) |
| Group III | ♂ | - (-) | - (-) | - (-) | - (-) | - (-) | — (-) | - (-) | - (-) |
| | ♀ | 1 (3) | 2 (7) | 1 (3) | - (-) | - (-) | — (-) | 2 (7) | - (-) |
| Group IV | ♂ | 3 (10) | 22 (71) | - (-) | - (-) | 3 (10) | 18 (58)[b,e] | - (-) | 1 (3) |
| | ♀ | 14 (48) | 16 (55) | 1 (3) | 6 (20) | 7 (24) | 10 (34)[b,d] | 1 (3) | 2 (7) |
| Group V | ♂ | - (-) | - (-) | - (-) | - (-) | - (-) | - (-) | - (-) | - (-) |
| | ♀ | 2 (7) | 1 (3) | 1 (3) | 1 (3) | - (-) | - (-) | 1 (3) | - (-) |
| Group VI | ♂ | - (-) | 1 (3) | - (-) | - (-) | - (-) | 1 (3) | - (-) | - (-) |
| | | 2 (7) | - (-) | - (-) | 2 (7) | - (-) | - (-) | - (-) | - (-) |

Malignant tumours of low incidence (No.).

Group I: Odontoblastoma of the lower jaw (1), adenocarcinoma of the lung (1), squamous cell carcinoma of the cutis (1).

Group II: Fibrosarcoma of the cutis (2), adenocarcinoma of the thyroid gland (1), squamous cell carcinoma of the lower jaw (1), odontoblastoma of the lower jaw (1), osteosarcoma (1), leiomyosarcoma of the uterus (1).

Group IV: Transitional cell carcinoma of the urinary tract (2), adenocarcinoma of the external auditory canal (2), adenocarcinoma of the prostata (1), squamous cell carcinoma of the lower jaw (1), fibrosacroma of the peritoneal cavity (1).

[a]Neurogenic sarcomas, astrocytomas, oligodendroblastomas, microgliomas and neurinomas of the *n. trigeminus* were subsumed to be malignant tumours of the nervous system. [aa]In group I four, in group II one and in group IV three animals had 2 neurogenic tumours. [b]Significantly different from group I; $P=0.0015$. [c]Significantly different from males of group I; $P=0.035$. [d]Females of groups I, II, IV significantly different from males of groups I, II and IV; $P=0.044$. [e]Significantly different from males of group I; $P=0.0008$. [f]Significantly different from males of group I; $P=0.04$.

$P=0.0015$) compared to the transplacental administration of nicotine (Group IV *vs.* Group II; $P=0.08$). Concomitant with the decreased occurrence of neurogenic tumours in rats treated with nicotine following birth, a shift in tumour type was observed from neurogenic sarcomas (70% instead of 85%) to gliomas (27% instead of 7%) compared with MNU-treated controls. Interestingly, the gender-specific imbalance in the occurrence of neurogenic tumours following MNU was diminished to an insignificant difference of 4% ($P=0.4$) in group II and of 14% ($P=0.25$) in group IV. The differences found in tumours of the mammary gland, however, were not significant according to an age-adjusted analysis of observed *versus* expected numbers of affected animals, although considerably less malignant mammary tumours appeared in both MNU and nicotine treated groups.

The regular injection of nicotine alone (groups III and V, respectively) had no overt influence on the survival or tumour yield in comparison to untreated controls.

### Discussion

The directly acting carcinogen MNU is well known to induce a variety of tumours, depending on the time and route of administration (Berger & Schmähl, 1986; IARC Monographs, 1978). In a first study on the modulation of MNU-induced tumorigenesis by nicotine in sexually premature rats, no apparent differences were obtained in the development of mammary tumours (Habs & Schmähl, 1984). This study was designed to assess the influence of nicotine on the same carcinogen during the perinatal stages of life. No influence on tumour development was exerted by nicotine alone (groups III and V *versus* group IV); this finding is in accordance with previously published data (Martin *et al.*, 1979). In comparison to a study by Alexandrov (1976), a higher dose level of MNU (30 mg kg$^{-1}$ instead of 20 mg kg$^{-1}$) and a different route of administration (p.o. instead of i.p.) were used. This resulted in a 20% increased yield of neurogenic tumours and the latency period was shortened by 250 days. The potential of MNU, however, in inducing neurogenic tumours was distinctly lower than that of ENU in parallel experiments (Habs & Schmähl, 1976; Ivankovic & Druckrey, 1968). At the same dose (mg kg$^{-1}$ body wt) ENU caused an incidence of neurogenic tumours of 92% in males and 90% in females. A gender-specific imbalance of neurogenic tumours was observed only at a dose of 10 mg kg$^{-1}$ ENU (62% incidence in males and 50% incidence in females), which was comparable to the results obtained in the present experiment. In contrast to this study, however, lifelong administration of nicotine (i.p.) resulted in 12% less neurogenic tumours in males, but in 16% more neurogenic tumours in females. Thus, nicotine did not significantly modulate the carcinogenic potential of ENU following transplacental administration.

Similar to that study, no change in the yield of urinary bladder tumours was obtained when nicotine was administered to *N*-butyl-*N*-(4-hydroxybutyl) nitrosamine-induced male F344 rats (Ito *et al.*, 1984). Unlike this result a significant enhancement in the number of *N*-methyl-*N*-nitro-*N*-nitroso-guanidine-induced tumours of the glandular stomach was detected in male albino rats following coadministration of nicotine (Gurkalo & Volfson, 1982). Another experiment which concentrated on the modification of *N*-(4-(5-nitro-2-furyl)-2-thiazolyl)formamide-induced tumours following administration of nicotine-derived metabolic products in male F344 rats, reported an increased total tumour load of rats, when these compounds were coadministered (LaVoie *et al.*, 1985). Interestingly, the incidence of urinary bladder tumours was significantly reduced. This is the only report which details an inhibitory effect of nicotine-related compounds on tumorigenesis, at least in one tissue. The results of the present study are even more surprising, since no complementary increase to the reduced occurrence of neurogenic tumours was detected in other tissues.

So far, no sufficient explanation is available for this inhibitory effect of nicotine on the development of neurogenic tumours. It might be speculated that the release of catecholamines caused by the administration of the neurotropic nicotine (Arqueros *et al.*, 1978) resulted in specific mechanisms of action leading to growth inhibition of neoplasms of the neurogenic tissue. In view of the reduced gender-specific differences in the observed tumour load following MNU and nicotine, the assumption of a hormonal influence cannot be excluded. A direct influence of nicotine on the decomposition or pharmacokinetics of MNU can be excluded, however, at least in group IV, where the administration of the modulating agent started one day after injection of the carcinogen. It is furthermore interesting to note that the seemingly protective effect of nicotine was observed independently of the fact, whether nicotine was administered before or after birth of the animals. The results, of course, do not mean that nicotine should be regarded as a beneficial or even harmless agent, since all other studies (Gurkalo & Volfson, 1982; Habs & Schmähl, 1984; Ito *et al.*, 1984; LaVoie *et al.*, 1985), including one on the same type of tissue (Habs & Schmähl, 1976), indicated an increased carcinogenic expression following this alkaloid or at least showed no apparent influence on experimental tumorigenesis. The observed unexpected modulation of carcinogenesis, however, warrants further investigations on compounds structurally related to MNU, such as alkylating cytotoxic agents.

The authors thank Prof. Dr D. Komitowsky, Institute of Pathology, German Cancer Research Center, for histologic evaluations and Mrs D. Theiss for careful technical assistance.

### References

ALEXANDROV, V.A. (1976). Some results and prospects of transplacental carcinogenesis studies. *Neoplasma*, **23**, 285.

ARQUEROS, L., NAQUIRA, D. & ZUNINO, E. (1978). Nicotine-induced release of catecholamines from rat hippocampus and striatum. *Biochem. Pharmacol.*, **27**, 2667.

BECKER, F., LITTLE, C. & KING, E (1968). Experimental studies on nicotine absorption in rats during pregnancy. *Am. J. Obstet. Gynecol.*, **100**, 957.

BECKER, F. & MARTIN, J (1971). Vital effects of chronic nicotine absorption and chronic hypoxic stress during pregnancy and the nursing period. *Am. J. Obstet. Gynecol.*, **110**, 522.

BERGER, M.R. & SCHMÄHL, D. (1987), Carcinogenicity of alkylating cytostatic drugs in animals. In *IARC Scientific Publications* (in press).

BOCK, F.G. (1980). Cocarcinogenic properties of nicotine: In *A Safe Cigarette?* Gori & Bock. Banbury Report 3. Cold Spring Harbor, New York.

BOYLAND, E. (1968). The possible carcinogenic action of alkaloids of tobacco and betel nut. *Planta Med. Suppl.*, **11**, 13.

DRUCKREY, H., PREUSSMANN, R. (1962). Zur Entstehung carcinogener Nitrosamine am Beispiel des Tabakrauchs. *Naturwissenschaften*, **49**, 498.

GURKALO, V.K. & VOLFSON, N. (1982). Nicotine influence upon the development of experimental stomach tumors. *Arch. Geschwulstforsch.*, **52**, 259.

HABS, M. & SCHMÄHL, D. (1976). Influence of five different postnatal lifelong treatments on the transplacental carcinogenicity of ethylnitrosourea in Sprague-Dawley rats. *Cancer Letters*, **2**, 93.

HABS, M. & SCHMÄHL, D. (1984). Influence of nicotine on nitrosomethylurea-induced mammary tumors in rats. *Klin. Wochenschr.*, **62** (Suppl. II), 105.

IARC MONOGRAPHS ON THE EVALUATION OF THE CARCINOGENIC RISK OF CHEMICALS TO HUMANS. (1978). Some Nitroso-compounds: *N*-methylnitrosourea, **17**, 227. IARC, Lyon.

ITO, N., FUKUSHIMA, S., SHIRAI, T., HAGIWARA, A. & IMAIDA, K. (1984). Drugs, food additives and natural products as promoters in rat urinary bladder carcinogenesis. *IARC Sci. Publ.*, **56**, 399.

IVANKOVIC, S. & DRUCKREY, H. (1968). Transplazentare Erzeugung maligner Tumoren des Nervensystems. I. Aethylnitrosoharnstoff (ANH) an BD-IX-Ratten. *Z. f. Krebsforsch.*, **71**, 320.

LAVOIE, E., SHIGEMATSU, A., RIVENSON, A., MU, B. & HOFFMANN, D. (1985). Evaluation of the effects of cotinine and nicotine-*N*-oxides on the development of tumours in rats initiated with *N*-(4-(5-nitro-2-furyl)-2-thiazolyl) formamide. *J. Natl Cancer Inst.*, **75**: 1075.

MARTIN, J., MARTIN, D., RADOW, B. & DAY, H. (1979). Life span and pathology in offspring following nicotine and methamphetamine exposure. *Exptl Aging Res.*, **5**, 509.

PETO, R., PIKE, M.C., DAY, N.E. & 6 others (1980). Guidelines for simple, sensitive significance tests for carcinogenic effects in long-term animals experiments. In *IARC Monographs on the evaluation of the carcinogenic risk of chemicals to humans* (Suppl. 2): Long-term and short-term screening assays for carcinogens, a critical appraisal, pp. 311, IARC: Lyon.

SCHMÄHL, D. & OSSWALD, H. (1968). Fehlen einer carcinogenen Wirkung von Cotinin bei Ratten. *Z. f. Krebsforsch.*, **71**, 198.

TOTH, B. (1982). Effects of long term administration of nicotine hydrochloride and nicotinic acid in mice. *Anticancer Res.*, **2**, 71.

TRUHAUT, R., DECLERCQ, M. & LOISILLIER, F. (1964). Sur les toxicites aigue et chronique de la cotinine, et sur son effet cancerigene chez le rat. *Path. Biol.*, **12**, 39.

YANYSHEVA, N.Y. & ANTONOMOV, Y.G. (1976). Predicting the risk of tumor occurrence under the effect of small doses of carcinogens. *Env. Health Perspect.* **13**, 95.